# Integrating Cardiology and Public Health: A Review of Population-Based Strategies for Cardiovascular Disease Prevention

**DOI:** 10.7759/cureus.106688

**Published:** 2026-04-08

**Authors:** Naresh Sen, Vikas Ashok Mishra, Naveed Mohsin, Rajeshwar Yadav, Komal Pukur Thekdi, Neerja Massey

**Affiliations:** 1 Department of Cardiology, Rama Medical College Hospital and Research Centre, Kanpur, IND; 2 Department of Cardiology, Superspeciality Hospital, Netaji Subhash Chandra Bose Medical College, Jabalpur, IND; 3 Department of Internal Medicine, Sher-i-Kashmir Institute of Medical Sciences Soura, Srinagar, IND; 4 Department of Cardiovascular and Thoracic Surgery, Institute of Medical Sciences, Banaras Hindu University, Varanasi, IND; 5 Department of Community Medicine, Dr. N.D. Desai Faculty of Medical Sciences and Research, Dharmsinh Desai University, Nadiad, IND; 6 Department of Medical Health, Auxiliary Nurse Midwife Training Centre, Bareilly, IND

**Keywords:** cardiology, digital health, prevention, public health, risk factors

## Abstract

This review examines population-based strategies for cardiovascular disease (CVD) prevention and evaluates how integration between cardiology and public health can facilitate effective and scalable approaches to reducing the global CVD burden. Despite major therapeutic advances, the incidence and mortality of cardiovascular disease continue to increase, particularly in low- and middle-income countries (LMICs), driven by demographic transitions, lifestyle changes, and socioeconomic inequities. Using a structured narrative synthesis, the review draws on epidemiological evidence, risk factor frameworks, policy interventions, and large-scale prevention initiatives to evaluate strategies across primordial, primary, and secondary prevention domains. The findings indicate that reductions in cardiovascular risk require approaches that extend beyond individual-level clinical management to address environmental, behavioral, and structural determinants of health. While interventions such as national screening programs, community-based strategies, and regulatory policies demonstrate measurable impact, gaps remain in evidence quality, health system integration, and equitable inclusion of vulnerable populations. The review highlights how alignment of clinical cardiology, public health systems, and multisectoral policies can improve prevention delivery and equity. It further emphasizes that strengthening primary care, advancing digital health technologies, and embedding prevention within broader social policy frameworks are essential for developing resilient cardiovascular prevention systems in resource-constrained settings.

## Introduction and background

Cardiovascular disease (CVD) is the leading cause of mortality globally and accounts for a significant share of deaths worldwide [1,2]. Despite advances in diagnostics, interventional procedures, and pharmacological therapies, the burden of CVD continues to rise, particularly in low- and middle-income countries (LMICs), where demographic transitions, urbanization, and lifestyle changes have increased exposure to cardiovascular risk factors [1,3,4]. Population ageing and the growing dominance of non-communicable diseases further contribute to clinical and economic pressures on health systems [5,6]. Although clinical cardiology has improved outcomes in individuals with established disease, its predominantly treatment-oriented focus remains inadequate to address cardiovascular risk at the population level [7]. The persistent high prevalence of modifiable risk factors, including hypertension, diabetes, dyslipidemia, tobacco use, and obesity, highlights the limitations of strategies confined to individual-level care [8].

Cardiovascular risk is shaped by interactions among biological, behavioral, and structural determinants. Factors such as income inequality, food environments, urban design, and access to preventive healthcare influence both exposure to risk and the capacity for sustained behavioral change [9,10]. A substantial proportion of cardiovascular morbidity and mortality is preventable through interventions that target these upstream determinants at scale [6,7]. Public health approaches emphasise prevention before disease onset and function through policy interventions, environmental modification, and health promotion [11,12]. These strategies complement clinical care by extending prevention beyond high-risk individuals to entire populations, thereby reducing the incidence of hypertension, diabetes, and atherosclerotic CVD through sustained, population-wide risk factor control [13,14]. Global initiatives, including non-communicable disease action plans and primary care-based cardiovascular programmes, underscore the importance of aligning clinical and population-level approaches to reduce the overall CVD burden [11,15].

**Figure 1:**
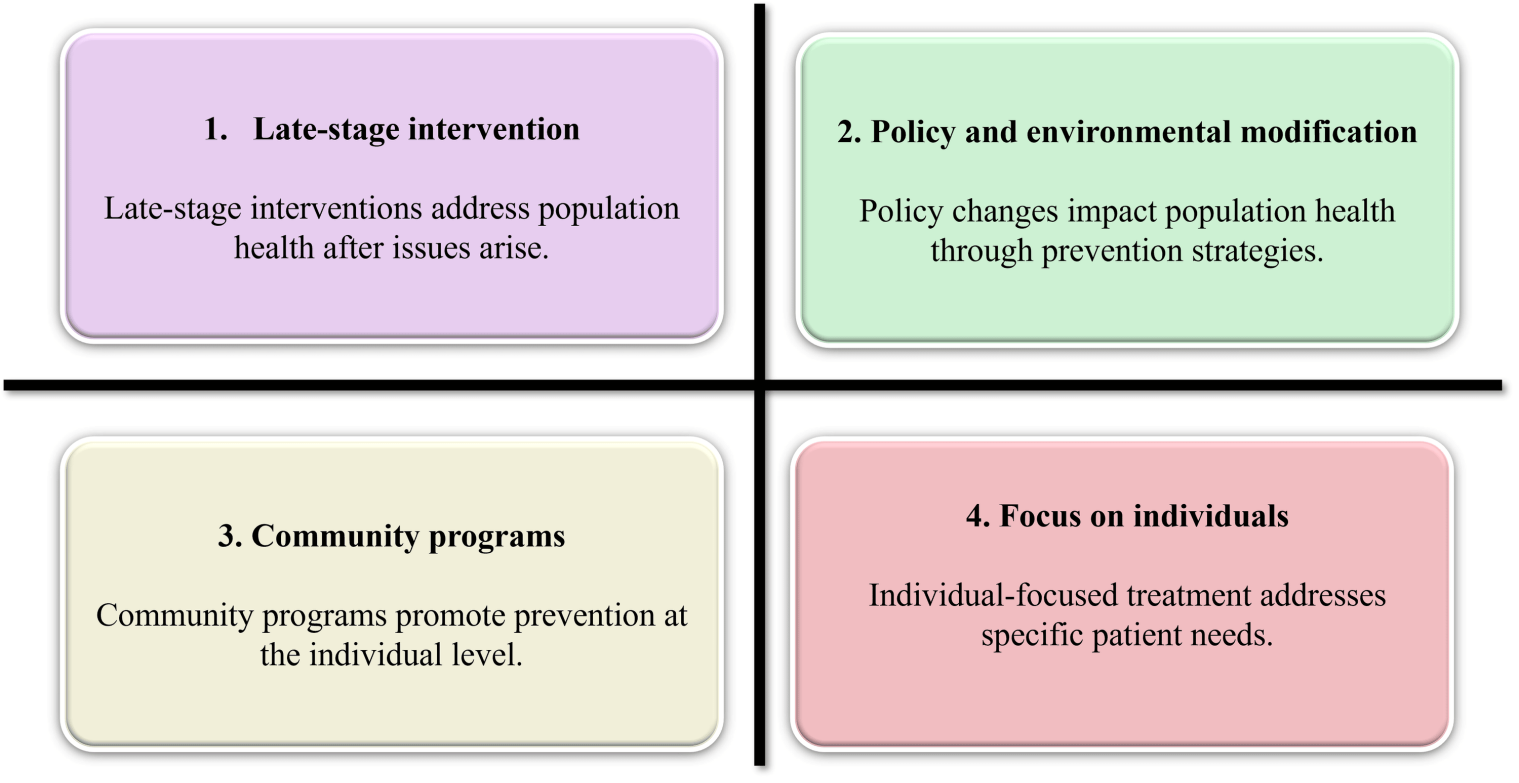
Conceptual Representation of Multilevel CVD Prevention Approaches Created by authors using Napkin AI (Los Altos, CA) CVD: cardiovascular disease

A coordinated approach linking clinical cardiology with public health systems enables simultaneous management of individual risk and modification of upstream determinants [16]. Clinical expertise in risk assessment and management can be complemented by public health capacities in implementation, surveillance, and policy development, enabling coordinated action across primordial, primary, and secondary prevention levels [17,18]. This alignment strengthens the delivery of screening services, optimization of risk factor control, and continuity of long-term management within both community and health system settings, leading to improved detection rates, better treatment adherence, and a reduced incidence of major cardiovascular events [15,19]. Such integrated models are particularly effective when embedded within primary care systems and supported by community-based delivery platforms, as illustrated in Figure 1.

Implementation of such integrated models remains constrained by systemic and contextual challenges. Fragmented health systems, limited resources, and workforce shortages limit the effectiveness, coordination, and scalability of prevention strategies across different healthcare settings [15,20]. Socioeconomic inequalities further exacerbate disparities in risk exposure, access to care, and health outcomes, particularly among disadvantaged and marginalized populations [21,22]. For example, lower-income populations experience higher exposure to unhealthy food environments and reduced access to preventive services, resulting in delayed diagnosis and poorer risk control [21,22]. These constraints highlight the need for context-sensitive, equity-oriented prevention models that prioritise accessibility, affordability, and inclusiveness.

Cardiovascular risk patterns continue to evolve, introducing additional complexity to prevention efforts. Sedentary behavior, unhealthy dietary patterns, psychosocial stress, and environmental exposures such as air pollution contribute to the changing epidemiology of CVD [23-25]. Digital health technologies offer new opportunities for risk monitoring and intervention delivery; however, their long-term effectiveness and equitable implementation remain uncertain [16,26,27]. Evidence suggests that digital tools can improve blood pressure monitoring and medication adherence. Still, their impact remains limited without systematic integration into routine clinical workflows, continuity of care, and structured follow-up mechanisms [26,27]. These developments require adaptive, multidisciplinary prevention strategies capable of addressing both established and emerging determinants of CVD within diverse population contexts [26,28].

Objectives of the review

This review aims to synthesize current evidence on population-based CVD prevention strategies across diverse populations and health system contexts, including high-income and low- and middle-income countries. It examines how clinical cardiology principles can be operationalised within public health systems to develop coordinated and scalable prevention pathways that improve risk detection, treatment adherence, and long-term disease control. The review evaluates the effectiveness and implementation challenges of large-scale interventions targeting major cardiovascular risk factors, drawing on evidence from established programmes and policy initiatives to assess their real-world impact on cardiovascular outcomes. It also identifies structural, governance, and equity-related gaps within existing collaborative models, particularly disparities in access to and continuity of care across socioeconomic groups. Finally, it outlines actionable directions for developing integrated and sustainable cardiovascular prevention systems grounded in clinical evidence and population health frameworks.

## Review

Methodology

Study Design

This study was conducted as a structured narrative review to examine the integration of cardiology and public health in CVD prevention. A narrative synthesis approach was chosen to accommodate heterogeneous evidence spanning epidemiology, prevention frameworks, policy interventions, implementation strategies, and clinical integration. This approach facilitates systematic, comparative, and interpretive analysis of diverse evidence sources rather than relying on quantitative aggregation of findings.

Search Strategy

A comprehensive literature search was conducted across PubMed, Scopus, Web of Science, Embase, and Google Scholar. The search covered publications from January 2015 to December 2025, with the final search carried out in December 2025. Both controlled vocabulary (e.g., MeSH terms) and free-text keywords were used, including “cardiovascular disease,” “cardiology,” “public health,” “primordial prevention,” “primary prevention,” “secondary prevention,” “population health,” “health systems,” and “policy interventions.” These terms were combined using Boolean operators (AND, OR), and search strategies were systematically adapted to the indexing frameworks of individual databases to ensure comprehensive retrieval. An example PubMed search string was: (“cardiovascular disease” AND “public health” AND “prevention” AND “health systems”). Reference lists of relevant articles and key reviews were also screened to identify additional studies, thereby improving the completeness of evidence retrieval and minimizing the risk of publication bias.

Selection Process and Scope

Study selection was conducted in two stages: title and abstract screening, followed by full-text review. Duplicate records were removed before screening. After the initial screening, a subset of studies was selected for full-text assessment based on relevance to CVD prevention, population health strategies, and the integration of clinical and public health systems. A total of 210 records were screened by title and abstract, of which 130 were excluded due to lack of relevance to the review objectives. Eighty full-text articles were assessed for eligibility, and 23 were excluded following detailed evaluation for reasons including a narrow clinical focus, non-cardiovascular outcomes, or insufficient alignment with the scope of the review. A total of 57 sources were included in the final narrative synthesis. Consistent with the narrative review design, study selection prioritized conceptual relevance, methodological robustness, and representation across diverse health system contexts rather than exhaustive inclusion. Two authors independently screened studies for relevance, and disagreements were resolved through discussion and consensus, thereby enhancing the reliability of selection and reducing subjective bias.

Eligibility Criteria

Inclusion criteria: Studies addressing CVD epidemiology, prevention strategies across primordial, primary, and secondary levels, or system-level and population-based interventions were included. Peer-reviewed original studies, systematic reviews, meta-analyses, and influential narrative reviews were considered.

Exclusion criteria: Studies focusing exclusively on narrowly defined clinical interventions without broader public health relevance, case reports, and studies unrelated to cardiovascular outcomes were excluded. Only English-language publications were included, which may introduce language bias and limit generalizability to non-English-speaking contexts.

Policy and Institutional Sources

In addition to peer-reviewed literature, policy documents, clinical guidelines, and institutional reports were included to capture system-level and implementation perspectives. These sources were identified through targeted searches of institutional websites, including the World Health Organization, the American Heart Association, and national public health agencies, as well as through reference tracking of included studies. Inclusion was based on relevance to cardiovascular prevention frameworks, policy implementation, and health system integration, allowing the incorporation of real-world program evidence alongside academic literature to enhance contextual relevance.

Data Extraction

Data were extracted using a structured descriptive approach, including study characteristics (year, setting, design), level of prevention (primordial, primary, or secondary), intervention type (policy, behavioral, clinical, or system-level), target population, and reported outcomes. For policy and institutional documents, emphasis was placed on regulatory approaches, implementation mechanisms, and population-level impact, enabling a systematic comparison of intervention types across varied health system contexts.

Quality and Bias Considerations

Formal risk-of-bias assessment tools were not applied due to the narrative design. Methodological quality was appraised qualitatively based on study design, sample representativeness, consistency of findings, clarity of outcome reporting, and disclosure of funding sources or conflicts of interest. Greater interpretive weight was assigned to systematic reviews, meta-analyses, and large-scale population studies, while findings from studies with methodological limitations were interpreted with caution, thereby strengthening the overall rigor and credibility of the synthesis despite methodological heterogeneity.

Synthesis Approach

The evidence was synthesized thematically using a conceptual framework based on levels of prevention (primordial, primary, and secondary) and domains of intervention (policy, environment, behavior, and health systems). Findings were compared across studies to identify consistent patterns, contextual variations, and implementation insights, including differences in effectiveness across population groups and healthcare settings. Policy and institutional sources were incorporated to contextualize empirical findings within real-world health systems. Quantitative pooling and meta-analysis were not performed, as the objective was conceptual synthesis rather than effect estimation, which is appropriate given the substantial heterogeneity in study designs, interventions, and outcome measures.

Epidemiological landscape of cardiovascular diseases

CVDs remain the leading cause of morbidity and mortality globally, with the overall burden continuing to rise despite advances in clinical cardiology [2]. While high-income countries have achieved reductions in CVD mortality through effective healthcare systems and improved risk factor control, low- and middle-income countries continue to experience a disproportionate increase in incidence and mortality rates [4]. This pattern reflects demographic transitions, increased life expectancy, and lifestyle changes associated with urbanization and economic development [7]. The coexistence of communicable and non-communicable diseases in these settings places additional strain on limited health system resources, further exacerbating inequities in prevention and care [5,6]. For example, delayed diagnosis and limited access to continuous treatment in resource-constrained settings contribute to higher rates of preventable cardiovascular events and deaths [5,6].

The distribution of CVD is strongly influenced by demographic and socioeconomic gradients [4,10]. Population ageing contributes significantly to the rising prevalence of ischaemic heart disease, heart failure, and cerebrovascular diseases [1,29]. Behavioral and environmental risk factors, including an unhealthy diet, tobacco use, psychosocial stress, and physical inactivity, are disproportionately concentrated in lower socioeconomic groups [30]. Limited access to healthcare contributes to delayed diagnosis and poorer clinical outcomes, including lower rates of hypertension control and reduced adherence to long-term pharmacological therapy. These patterns indicate that cardiovascular risk is shaped not only by biological processes but also by broader social and environmental determinants [10].

**Figure 2:**
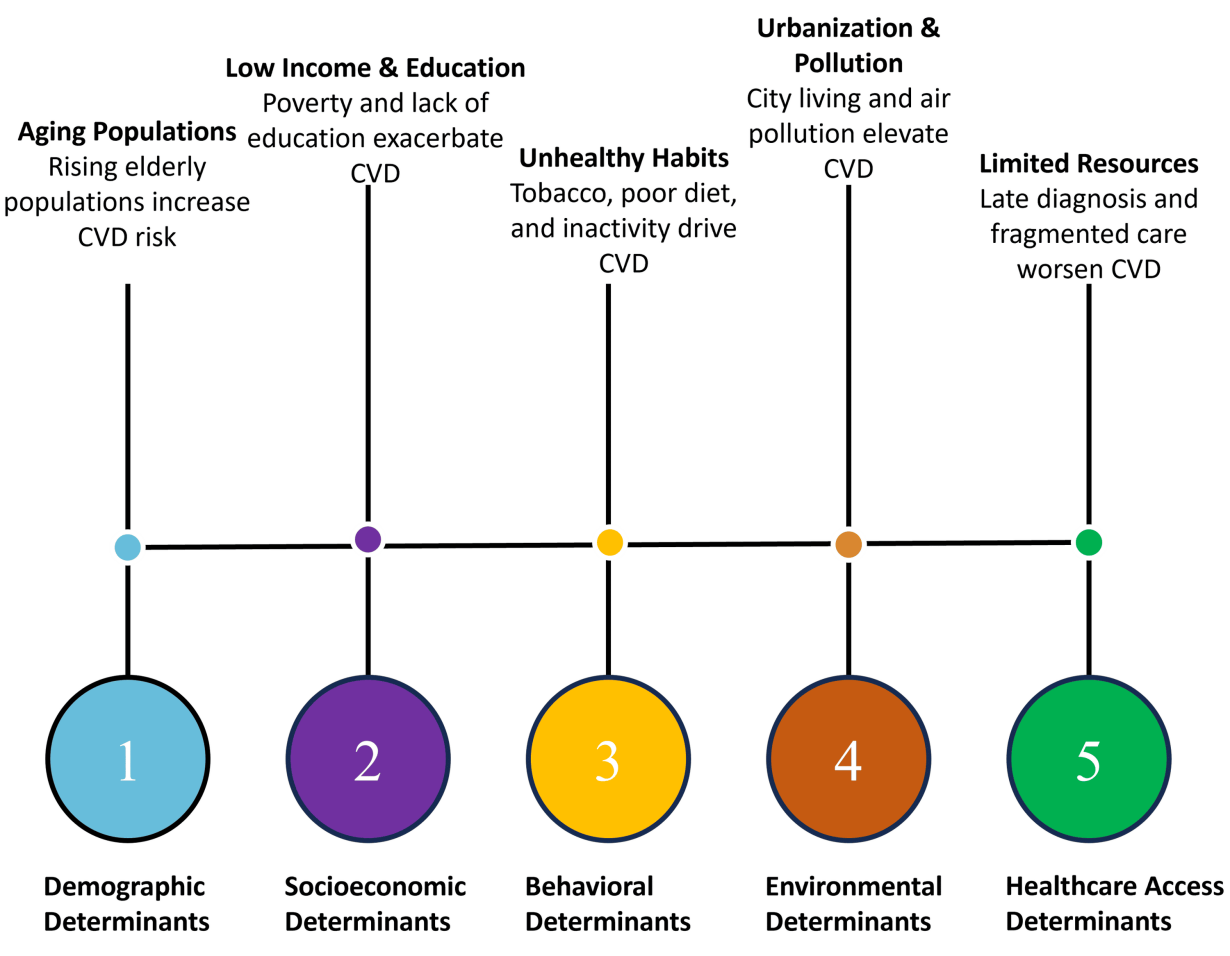
Determinants of CVD Created by authors using Napkin AI CVD: cardiovascular disease

This epidemiological context underscores the need for comprehensive preventive strategies operating across primordial, primary, and secondary levels of prevention, each targeting distinct stages of disease development [31]. Addressing these gradients requires coordinated interventions that combine risk factor modification with improved access to screening and long-term care. The distribution of CVD is shaped by key determinants, including demographic, socioeconomic, behavioral, environmental, and healthcare access factors, as shown in Figure 2.

Risk factor framework: linking clinical cardiology with population health

Understanding the spectrum of risk factors underlying CVD is essential for developing targeted and contextually relevant prevention strategies across clinical and population health settings [7,31]. Hypertension, diabetes, dyslipidemia, tobacco use, and obesity remain the principal drivers of cardiovascular morbidity, with their impact amplified by gaps in detection, treatment, and long-term management [8]. Hypertension is a major contributor to stroke and ischemic heart disease, but remains inadequately controlled across many health systems [11]. Diabetes accelerates vascular damage through chronic metabolic dysregulation, particularly in populations undergoing rapid nutritional and lifestyle transitions [12]. Dyslipidemia promotes atherosclerotic plaque formation, especially in dietary contexts characterized by high intake of saturated and trans fats [32]. Tobacco use induces endothelial injury and prothrombotic states, while obesity acts as an upstream driver by promoting insulin resistance, hypertension, and lipid abnormalities [8,31].

These risk factors frequently cluster within individuals, leading to cumulative increases in cardiovascular risk. For example, the coexistence of hypertension and diabetes substantially elevates the likelihood of macrovascular complications [3], resulting in higher rates of myocardial infarction, stroke, and heart failure. Environmental and structural determinants further modify this risk profile at the population level [12]. Exposure to fine particulate air pollution contributes to systemic inflammation and endothelial dysfunction, increasing the incidence of ischemic cardiovascular events [33]. Psychosocial stress related to economic instability influences neuroendocrine pathways, contributing to hypertension and metabolic dysregulation [34]. These exposures are unequally distributed and intersect with socioeconomic disadvantage, resulting in higher cumulative risk among vulnerable populations due to both increased exposure and reduced access to preventive care [1,9]. This unequal distribution of risk factors explains disparities in cardiovascular outcomes across socioeconomic and geographic groups.

This multilevel risk structure requires prevention strategies that extend beyond individual-level management. Clinical cardiology provides tools for risk stratification and evidence-based management [11], but their population impact depends on system-level implementation, including screening, continuity of care, and adherence support [4]. Integrating these clinical approaches with population-level interventions enables the simultaneous control of individual risk factors and the reduction of upstream exposures, which is necessary to achieve sustained reductions in cardiovascular incidence and mortality [20].

Primordial prevention: policy-level interventions

Primordial prevention aims to prevent the emergence of cardiovascular risk factors by addressing underlying social, environmental, and structural determinants of health [10,35]. Regulatory interventions have demonstrated effectiveness in shaping population-level behaviors. Tobacco control policies, including taxation, advertising restrictions, smoking bans, and plain packaging, have contributed to significant reductions in smoking prevalence and subsequent cardiovascular morbidity and mortality [7,36]. These measures illustrate the capacity of policy-level interventions to modify behavioral risk patterns before their clinical manifestation [14]. Nutrition policies similarly influence population dietary patterns. Interventions such as the elimination of industrial trans fats, mandatory nutrition labeling, and sodium reduction programs have improved cardiovascular risk profiles at scale, including reductions in serum cholesterol levels and blood pressure across populations [37]. Food reformulation initiatives further demonstrate that alignment between industry practices and public health policies can support sustained improvements in dietary behavior [32].

Built environment and urban planning interventions are central to influencing physical activity and sedentary behavior [10,13]. Strategies including the development of walkable urban spaces, expansion of green areas, and improved cycling and pedestrian infrastructure promote routine physical activity, particularly in urban settings characterized by sedentary lifestyles [2,13]. Evidence from population-level studies indicates that such environmental modifications are associated with increased physical activity levels and lower incidence of obesity and hypertension [2,13]. Modifications to food environments, especially in underserved communities, enhance access to affordable, nutritious foods while limiting the availability of energy-dense, nutrient-poor options [38]. These upstream interventions operate at the population level to reduce exposure to cardiovascular risk factors before clinical disease onset, thereby lowering long-term disease incidence and reducing the overall healthcare burden [10,31].

Primary prevention: community-based cardiovascular risk reduction

Primary prevention targets the modification of cardiovascular risk factors before the onset of clinically manifest disease through community-based and population-level interventions that enhance early detection and risk reduction [31,35]. Community-based strategies extend prevention beyond clinical settings by embedding interventions within everyday social and environmental contexts, thereby improving reach among populations that underutilize formal healthcare services [16,20]. Community health workers enable early identification of risk factors such as hypertension, unhealthy diet, and tobacco use, while supporting behavioral change through culturally appropriate counseling and follow-up [15,39].

This decentralized model is particularly effective in settings with limited healthcare access, where it improves detection and facilitates continued engagement with preventive care [19,39]. Blood pressure screening is a high-impact strategy due to the asymptomatic nature of hypertension and its strong association with major cardiovascular events [1,39]. Community-based screening in workplaces, schools, markets, and transport hubs increases detection among otherwise unreached populations and enables referral into care pathways. Its effectiveness depends on linkage to care and early management, as timely treatment initiation reduces major cardiovascular events, including myocardial infarction and stroke [39].

Lifestyle interventions are most effective when delivered through structured, context-specific platforms that support sustained behavioral change. School-based programs establish early dietary and physical activity patterns that persist into adulthood [36]. Workplace interventions provide scalable opportunities for risk reduction through improvements in food environments, promotion of physical activity, and stress management initiatives [40]. Community institutions, including religious and cultural organizations, enhance uptake by leveraging existing social networks and trust structures [10].

Health literacy is a critical enabling factor, as improved understanding of risk factors is associated with better adherence to preventive behaviors [19]. Higher health literacy is also linked to improved medication adherence and blood pressure control in community-based programs [19]. Interventions incorporating culturally tailored communication demonstrate greater effectiveness by aligning strategies with local norms, thereby improving participation and sustaining long-term behavior change [15,20].

Secondary prevention through public health integration

Secondary prevention focuses on the early identification and management of cardiovascular risk factors to prevent progression to clinically manifest disease [31,41]. Its effectiveness depends on the integration of clinical pathways within public health systems to ensure coordinated screening, diagnosis, and long-term management. This integration enables consistent screening, timely diagnosis, and sustained management across populations while addressing gaps such as fragmented care and inadequate follow-up [17,20,41]. National screening programs for hypertension, diabetes, and dyslipidemia are most effective when embedded within organized primary care systems and supported by policy frameworks, as this approach improves detection and facilitates timely initiation of treatment [8].

The impact of secondary prevention is determined by continuity of care from detection to long-term management. While screening identifies high-risk individuals, outcome reduction depends on sustained adherence to pharmacological therapy, lifestyle modification, and regular monitoring [18]. Public health systems reinforce this continuum through structured counseling, community-based follow-up, and policies that improve affordability and access to essential medications, thereby reducing loss to follow-up and improving long-term risk control [16,42]. Sustained adherence to antihypertensive, lipid-lowering, and glucose-lowering therapies is associated with significant reductions in recurrent cardiovascular events and mortality [18]. Coordination between primary care, specialist services, and community programs further strengthens continuity of care, ensuring effective transitions across different levels of the health system without interruption in management [18].

Digital health technologies enhance this continuum by enabling ongoing monitoring and engagement beyond clinical settings [26]. Mobile applications, remote monitoring devices, and telehealth platforms facilitate tracking of blood pressure and glucose levels while providing reminders and personalized feedback [42,43]. These tools improve adherence and generate data that support population-level planning and resource allocation [20,44]. For example, remote monitoring programs have been shown to improve blood pressure control and patient engagement when combined with clinical follow-up systems [42,43]. Their effectiveness is greatest when integrated within existing health systems, as isolated digital interventions show limited impact without linkage to clinical care and follow-up structures [20,26].

Integrated secondary prevention models operate through a defined pathway: systematic screening, effective linkage to care, sustained adherence, and continuous monitoring. Disruptions at any stage reduce effectiveness, whereas coordinated systems significantly lower disease progression and recurrent cardiovascular events [18,45]. This pathway highlights that secondary prevention is a continuous, system-level process requiring coordination across clinical, community, and policy domains. As illustrated in Table 1, secondary prevention depends on aligned clinical and community-level interventions that reinforce detection, adherence, and long-term risk management.

**Table 1:** Key Components of Secondary CVD Prevention and Their Public Health Integration CVD: cardiovascular disease

Component of Secondary Prevention	Core Activities	Role of Public Health Integration	Impact on CVD Outcomes	References
Early Detection and Screening	Standardized screening for hypertension, diabetes, and dyslipidemia	National programs ensure equitable access and systematic risk identification	Facilitates early diagnosis and reduces progression to major cardiovascular events	[11]
Continuity of Care and Follow-up	Structured monitoring, referral pathways, and case management	Coordination across primary, secondary, and community care systems	Improves long-term risk factor control and reduces complications due to care discontinuity	[17]
Medication Adherence Support	Long-term management with antihypertensives, statins, and glucose-lowering therapies	Counseling and policies improving affordability and access to essential medicines	Enhances adherence and reduces recurrent cardiovascular events and mortality	[30]
Lifestyle Modification Interventions	Behavioral counseling for smoking cessation, diet, and physical activity	Community-based programs and health literacy initiatives supporting sustained behavior change	Reduces modifiable risk factors and supports long-term cardiovascular risk reduction	[24]
Digital Health Technologies	Use of mHealth, telehealth, and remote monitoring tools	Integration with public health systems to support continuous monitoring and patient engagement	Improves monitoring, adherence, and early risk detection	[26]
Integrated Care Systems	Multidisciplinary coordination and shared-care models	Integration of cardiology, primary care, community health workers, and public health systems	Reduces care fragmentation and improves continuity across the prevention pathway	[18]

Integrating cardiology with public health systems

The integration of cardiology and public health provides a systems-level approach to strengthening CVD prevention by linking clinical care with population-based strategies [16,20]. This model combines clinical expertise in risk assessment and management with public health capacities in implementation, surveillance, and policy development [31,46]. Effective integration requires coordination among cardiologists, primary care providers, community networks, and public health institutions to deliver prevention across the continuum of care [15,18]. Shared-care models are central to operationalizing integration. In these models, primary care providers manage stable patients using standardized protocols, with referral to cardiologists for complex cases [17].

This approach optimizes resource utilization, reduces unnecessary specialist consultations, and ensures continuity of care while enabling broader population coverage through decentralized service delivery. Integration is further supported by interoperable electronic health records and clearly defined referral pathways, which enable efficient information exchange and reduce fragmentation across healthcare systems [26]. Evidence from integrated care models indicates improvements in blood pressure control, adherence to guideline-directed therapy, reductions in avoidable hospitalizations, and lower rates of recurrent cardiovascular events, demonstrating measurable improvements in clinical outcomes and health system efficiency [18].

Implementation at scale is demonstrated through national and global initiatives [11,15]. The United Kingdom’s National Health Service (NHS) illustrates how coordinated primary care, specialist services, and population health systems improve cardiovascular outcomes, including hypertension control and reduced hospital admissions for cardiovascular events [47]. In India, the National Programme on Prevention and Control of Cancer, Diabetes, Cardiovascular Diseases and Stroke (NPCDCS) demonstrates the feasibility of embedding cardiology-informed protocols within large-scale public health systems, particularly in resource-constrained settings where access to specialist care is limited [48]. The World Health Organization HEARTS programme further supports integration through standardized treatment algorithms, capacity-building initiatives, and simplified management tools for diverse healthcare settings [11].

**Figure 3:**
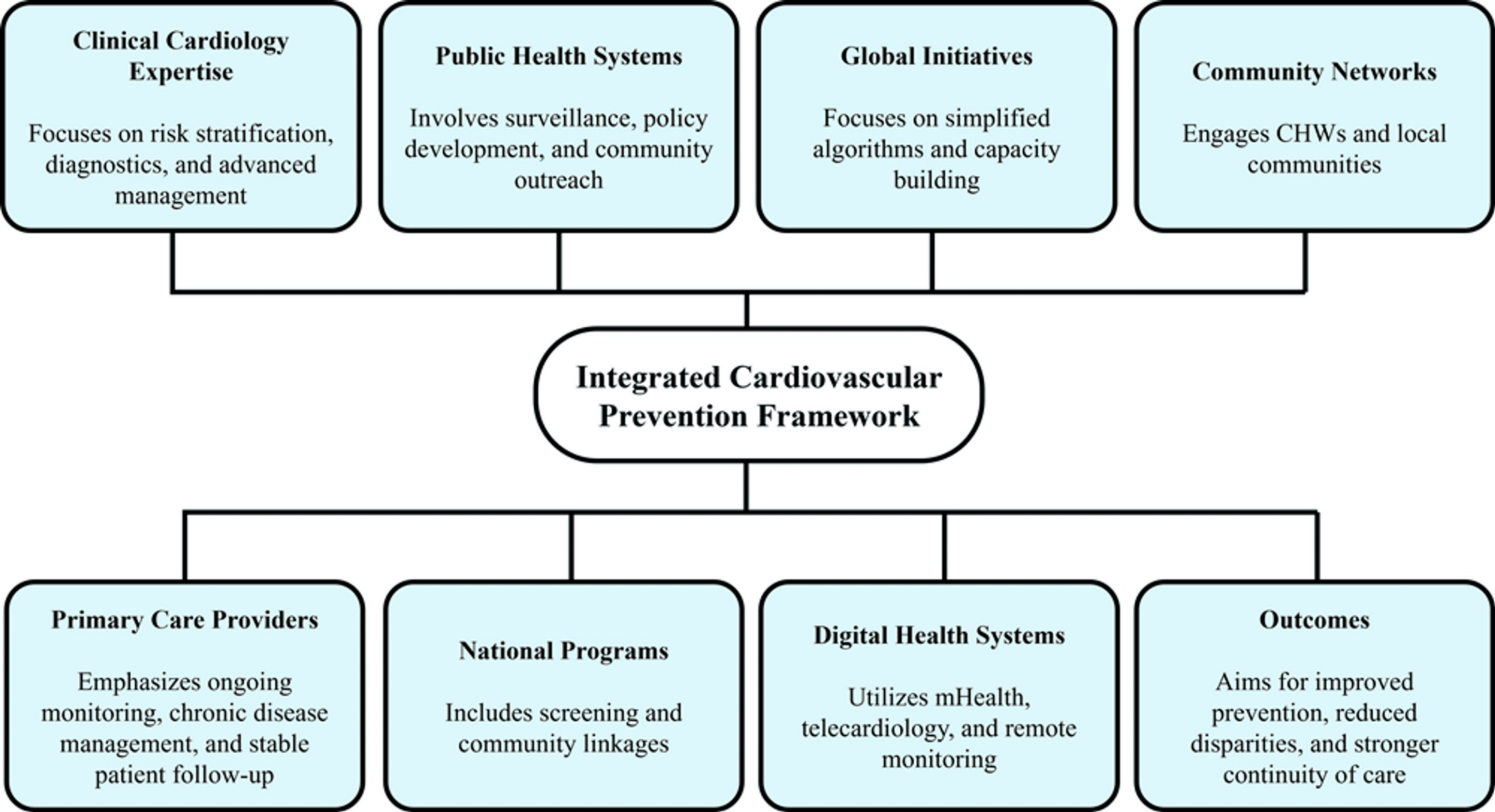
Integrated Cardiology-Public Health Prevention Framework Created by authors using Napkin AI

These models demonstrate that integrated systems enhance the scalability, equity, and sustainability of cardiovascular prevention strategies, particularly when supported by strong primary care infrastructure and policy alignment [49]. While high-income models such as the NHS demonstrate effectiveness through well-developed infrastructure and coordinated care pathways, programs in low- and middle-income settings, including NPCDCS and WHO HEARTS, rely more heavily on simplified protocols and task-shifting strategies to achieve scalability under resource constraints, highlighting differences in implementation approaches across health system contexts. By aligning clinical management with population-level implementation, integrated approaches contribute to sustained reductions in cardiovascular burden [18,20]. The interaction between cardiology, primary care, public health systems, digital platforms, and community networks is illustrated in Figure 3.

Socioeconomic and behavioral determinants in CVD prevention

Socioeconomic and behavioral determinants influence cardiovascular health by shaping patterns of risk exposure, healthcare access, and adherence to preventive interventions [10,50]. Income operates as a fundamental determinant by structuring living conditions, food environments, and access to healthcare services, thereby influencing both the development and management of cardiovascular risk factors [51]. Lower socioeconomic status is associated with greater exposure to environments that limit physical activity and access to healthy foods, resulting in higher cardiometabolic risk, including increased prevalence of obesity, hypertension, and diabetes [13].

Financial constraints also delay engagement with screening and preventive services, leading to later detection and poorer risk control, which contributes to more advanced disease and complications at presentation [52]. Educational attainment further modifies outcomes through its effect on health literacy and the capacity to engage with preventive care. Higher education levels are associated with improved adherence to lifestyle and therapeutic interventions, whereas limited literacy restricts effective healthcare utilization [49,53]. Social capital, reflected in community cohesion and trust, enhances participation in preventive programs and supports continuity of care, thereby improving outcomes across prevention levels [52].

These determinants interact across contexts rather than acting independently. Geographical variation illustrates this interaction, as urban environments are associated with increased exposure to air pollution and psychosocial stressors, while rural settings face workforce shortages and limited access to structured prevention services [13,45]. Such differences influence both exposure to risk and the capacity for intervention delivery. Behavioral factors, including physical inactivity, unhealthy diet, and chronic stress, represent proximal expressions of these broader determinants and are key targets for intervention [34,37]. Behavioral economics further explains that these behaviors are shaped by environmental cues and decision contexts rather than purely individual choice [53]. For example, limited availability of healthy food options and the widespread presence of energy-dense foods can reinforce unhealthy dietary behaviors despite awareness of health risks.

Interventions such as default enrollment in screening programs, incentive-based strategies, and modification of food environments operate by altering these contexts, thereby enabling behavior change at scale [35]. Social and cultural factors also influence engagement with prevention. Gender norms and social expectations affect healthcare utilization, with structural barriers limiting access among women in some settings and behavioral norms reducing service uptake among men [49]. Cultural beliefs and dietary practices can affect adherence to recommended interventions, particularly when health messages are not aligned with local practices. These intersecting influences indicate that cardiovascular prevention requires context-specific strategies integrating socioeconomic, behavioral, and cultural determinants into implementation frameworks [35,41]. Among these determinants, food environment and access to preventive healthcare services emerge as the most immediately modifiable factors, as they can be directly influenced through policy interventions and health system strengthening, whereas broader socioeconomic inequalities require longer-term structural change.

Evaluation of population-based cardiovascular disease prevention programs

Population-based cardiovascular prevention initiatives require systematic evaluation to assess effectiveness, scalability, and sustainability [31,50]. Core indicators include reductions in disease incidence, cost-effectiveness, population coverage, and adherence to recommended interventions [54]. Among these, incidence reduction provides the most direct measure of impact, reflecting decreases in hypertension, diabetes, and cardiovascular events [55]. Cost-effectiveness is critical in resource-constrained settings, where prioritization depends on maximizing health gains per unit of investment. Population coverage and adherence together determine real-world effectiveness, as high coverage without sustained engagement limits outcomes, while strong adherence in small populations restricts overall impact [19,28]. Therefore, optimal programs require both broad population reach and sustained individual engagement to reduce cardiovascular burden.

Evidence from large-scale programs demonstrates that effectiveness depends on implementation context and system integration [7]. The Finnish North Karelia Project achieved substantial reductions in cardiovascular risk through sustained, multisectoral action, including food system reform, tobacco control, and community mobilization, resulting in marked declines in cholesterol levels, smoking prevalence, and coronary mortality [36,56]. In contrast, global initiatives such as the WHO Global HEARTS programme emphasize standardization and scalability through simplified clinical protocols and primary care strengthening, improving hypertension detection and management in primary care settings [7,11]. The US Million Hearts Initiative combines clinical quality improvement with public health strategies, demonstrating improvements in hypertension control and tobacco cessation at a national scale [15,45,57].

Comparative analysis indicates that long-term success is influenced by sustained implementation, strong health system integration, and alignment between policy interventions and community engagement strategies. Notably, the North Karelia Project demonstrates the effectiveness of long-term population-wide behavioral and policy interventions, whereas programs such as WHO HEARTS and Million Hearts emphasize standardized, system-based approaches, reflecting differing strategies for achieving impact across varied health system contexts. Programs with long-term political commitment and stable financing demonstrate greater reductions in population risk, whereas short-term or fragmented initiatives show limited scalability and weaker impact. Key domains of evaluation, major initiatives, and determinants of program effectiveness are summarized in Table 2.

**Table 2:** Evaluation of Population-Based CVD Prevention Programs CVD: cardiovascular disease

Evaluation Domain	Key Elements	Summary of Evidence from Literature	Impact on CVD Outcomes	References
Evaluation Metrics	Incidence reduction, cost-effectiveness, population coverage, adherence	Standardized indicators enable consistent assessment of effectiveness, scalability, and efficiency across settings	Identifies effective strategies and implementation gaps across populations	[14]
North Karelia Project (Finland)	Community mobilisation, dietary reform, and tobacco control	Sustained multisectoral interventions reduced cholesterol levels, smoking prevalence, and coronary mortality	Demonstrates long-term population-level reduction in cardiovascular risk and mortality	[56]
WHO HEARTS Programme	Standardized treatment protocols, essential medicines, and capacity building	Strengthens primary care systems and improves continuity of prevention and management	Improves hypertension detection, treatment coverage, and risk factor control at scale	[11]
US Million Hearts Initiative	ABCS framework, sodium reduction, clinical quality improvement	Coordinated national strategies improved hypertension control and tobacco cessation	Reduces major cardiovascular events through improved risk factor management	[37]
Determinants of Programme Success	Political commitment, sustainable financing, and community engagement	Stable governance and long-term funding support sustained implementation and effectiveness	Enables sustained population-level risk reduction and programme impact	[5]
Common Barriers	Weak surveillance, socioeconomic inequities, low participation, and limited system capacity	Structural and data limitations constrain participation and scalability	Reduces programme reach and effectiveness, increasing outcome disparities	[41]

Challenges and barriers in integrating cardiology with public health

The integration of cardiology and public health is constrained by organizational, financial, and systemic barriers that limit implementation and scalability [20]. Health system fragmentation and workforce limitations are key constraints, directly affecting service delivery and continuity of care. Shortages of cardiologists, primary care providers, nurses, and health promotion personnel, particularly in low- and middle-income countries, reduce capacity for coordinated prevention and management, leading to gaps in screening, delayed treatment, and inadequate follow-up [5,7]. Fragmented systems further create discontinuities in care and inefficient resource utilization, weakening integrated prevention models [17,39]. Financial constraints reinforce these limitations, as cardiovascular prevention competes with immediate health priorities, resulting in underinvestment in long-term strategies [3,5]. Short-term funding cycles restrict scalability, while limited recognition of the economic benefits of prevention reduces policy prioritization [28]. These interacting barriers reduce program effectiveness and long-term sustainability.

Behavioral and contextual factors further influence program effectiveness. Sustained adherence to treatment, monitoring, and lifestyle modification remains challenging in settings characterized by socioeconomic disadvantage, limited health literacy, and competing priorities [10,15,19]. These challenges are more pronounced among marginalized populations, where structural inequities increase both risk exposure and barriers to care, resulting in lower adherence and poorer risk control [21]. A key systemic issue is the separation between cardiology and public health systems [20]. Weak communication, incompatible information systems, and limited data sharing hinder coordinated care and reduce efficiency [20,28]. This limits surveillance and integration of clinical insights into public health planning. Addressing these challenges requires investment in interoperable systems, cross-disciplinary collaboration, and governance structures that align clinical and population health objectives [17,20].

Study limitations and recommendations

The current evidence base on population-level cardiovascular disease prevention is characterized by methodological heterogeneity and limited contextualization, reducing comparability and interpretability of findings. Variability in study design, implementation quality, and measurement approaches limits synthesis across settings, making it difficult to compare intervention effectiveness across populations and health systems. Many interventions lack long-term follow-up, limiting assessment of sustained effectiveness and impact on outcomes such as recurrent cardiovascular events and mortality. Evidence is disproportionately derived from high-income settings, resulting in underrepresentation of low-income and vulnerable populations. Additional limitations include the absence of standardized outcome measures and limited evaluation of digital health interventions, particularly regarding scalability, affordability, and equity. These gaps constrain the development of generalizable prevention frameworks. Among these limitations, the underrepresentation of low- and middle-income populations represents the most critical gap, as it restricts the applicability of prevention strategies to settings where the burden of cardiovascular disease is highest.

Future efforts should prioritize rigorous, standardized, and equity-oriented research. Strengthened interdisciplinary collaboration among cardiologists, epidemiologists, public health practitioners, and policymakers is necessary to develop coherent prevention strategies. Greater investment in community- and school-based interventions, alongside responsible scaling of digital health technologies, can enhance implementation when integrated with primary care systems. Improved surveillance and real-time monitoring are required to track evolving risk patterns and identify high-risk populations. Multisectoral policies addressing social determinants remain essential for targeting upstream drivers of cardiovascular disease. Longitudinal studies in underrepresented populations are important for evaluating long-term effectiveness, cost-efficiency, and equity across diverse settings.

## Conclusions

This review demonstrates that the integration of cardiology and public health provides a systems-oriented framework for reducing the global burden of CVD by addressing clinical, behavioral, and structural determinants of risk. It synthesizes epidemiological evidence, policy interventions, community-based strategies, and shared-care models within a unified framework that links individual risk management to population-level system design, highlighting how coordinated approaches improve risk detection, treatment adherence, and long-term cardiovascular outcomes. Effective prevention requires alignment between clinical expertise, public health infrastructure, primary care systems, and policies targeting social and environmental determinants of health, as these components collectively enable comprehensive risk reduction across populations. Despite demonstrated benefits, implementation remains constrained by fragmented health systems, limited resources, and persistent inequities, which limit scalability and consistent delivery of preventive interventions. A coordinated approach that prioritizes scalability, equity, and system integration is therefore essential to achieve sustainable, population-level cardiovascular prevention across diverse settings, with an emphasis on strengthening primary care, improving access to essential services, and addressing social determinants of health.
